# The Stock Exchange and the Hospitals

**Published:** 1895-10-26

**Authors:** 


### THE STOCK EXCHANGE AND THE HOSPITALS.

### HOW THE £50,000 WAS COMPLETED.

Messrs. Burdett and Harris and Messrs. Pim, Vaughan, and Co. having undertaken to bring the special claims of the hospitals under the notice of the members of the Stock Exchange, issued the following notice :—" Special S. E. Fund to raise the Hospital Sunday Collection in 1895 to £50,000: The Lord Mayor, as the treasurer, in conjunction with The Hospital newspaper, owing to the pressing needs of the hospitals this year, is making a supreme effort to raise the total collection to £50,000. So far about £46,000 has been received, and if £4,000 can be raised in the House immense benefit will be conferred upon many poor suffering Londoners, who may otherwise be unable to obtain the hospital relief which they need. If the total collection is once raised to £50,000 experience shows that it will continue at about that amount each year, so that something like an adequate and permanent addition will be made to the resources of the hospitals." As a result of this appeal, the sum of £3,400 has been subscribed, and the Metropolitan Sunday Fund raised in 1895 to £50,000.

### LIST OF SUBSCRIPTIONS.

| | £ | s. |
|---|---|---|
| A. J. W. | 105 | 0 |
| Ansell, Mankiewicz, and Tallerman | 52 | 10 |
| A. M. | 52 | 10 |
| Burdett and Harris | 50 | 0 |
| Hyam Bros. | 50 | 0 |
| Whitehead and Hilder | 50 | 0 |
| Pim, Vaughan, and Co. | 50 | 0 |
| Heseltine and Co. | 50 | 0 |
| De la Bere and Bellairs | 50 | 0 |
| Simpson Bros. | 50 | 0 |
| Hilder and Co. | 50 | 0 |
| A. Hirsch | 50 | 0 |
| Buckler, Norman, & Co. | 50 | 0 |
| Renton Bros. | 50 | 0 |
| Wedd, Jefferson, & Co. | 50 | 0 |
| Ricardo and Robertson | 50 | 0 |
| Ricardo and Co. | 50 | 0 |
| J. L. | 50 | 0 |
| S. Kennedy and Co. | 50 | 0 |
| R. Nivison and Co. | 50 | 0 |
| H. H. B. | 50 | 0 |
| A. M. H. | 50 | 0 |
| Geo. Cawston | 50 | 0 |
| Anon | 50 | 0 |
| Cazenove and Akroyds | 50 | 0 |
| A. Haes | 26 | 5 |
| L. Upton | 26 | 5 |
| Foster & Braithwaite | 26 | 5 |
| L. Messel and Co. | 26 | 5 |
| Borthwick, Wark, & Co. | 26 | 5 |
| Steer, Lawford, & Co. | 26 | 5 |
| Kennedy & Robertson | 26 | 5 |
| Linton, Clarke, & Co. | 26 | 5 |
| Morris, Hurst, & Puckle | 25 | 0 |
| Michels, Prentice, & Co. | 25 | 0 |
| Gundry, Mills, and Co. | 25 | 0 |
| Marnham and Co. | 25 | 0 |
| S. Bontein & Co. | 25 | 0 |
| Reginald Barker | 25 | 0 |
| R. W. Inglis | 25 | 0 |
| Panmure Gordon & Co. | 25 | 0 |
| F. C. Stoop and Co. | 25 | 0 |
| Christopherson & Tonge | 25 | 0 |
| Marzetti and Co. | 25 | 0 |
| Vertue, Lubbock, & Co. | 25 | 0 |
| W. E. Marshall | 25 | 0 |
| A. G. Schiff and Co. | 25 | 0 |
| Louis Cohen and Sons | 21 | 0 |
| Hilton, Gibbes, and Smith | 21 | 0 |
| Hore and Doidge | 21 | 0 |
| Price and Pott | 21 | 0 |
| Laing and Cruickshank | 20 | 0 |
| Löwy and Campbell | 20 | 0 |
| Stoneham and Co. | 20 | 0 |
| P. L. Smith | 20 | 0 |
| C. A. Grenfell | 20 | 0 |
| Marten and Christopherson | 20 | 0 |
| J. De Pass | 20 | 0 |
| J. T. Dadson | 20 | 0 |
| Crews & Lichtenstadt | 20 | 0 |
| Ed. Coates and Co. | 20 | 0 |
| Simon Symons and Co. | 20 | 0 |
| Bone, Oldham, and Mordaunt | 10 | 10 |
| Pritchett and Young | 10 | 10 |
| Tapp and Landau | 10 | 10 |
| A. Thomson and Co. | 10 | 10 |
| Holland, Balfour, & Co. | 10 | 10 |
| J. G. Williamson | 10 | 10 |
| Handcock and Webb | 10 | 10 |
| Milln and Robinson | 10 | 10 |
| Conrad Wilkinson | 10 | 10 |
| Hugh Wyatt | 10 | 10 |
| F. Armstrong | 10 | 10 |
| Baker, Mason, and Co. | 10 | 10 |
| G. Kitchin and Co. | 10 | 10 |
| F. and N. Durlacher | 10 | 10 |
| G. P. Howard | 10 | 10 |
| R. S. Johnstone | 10 | 10 |
| Robson Bros. | 10 | 10 |
| Tom Nickalls and Co. | 10 | 10 |
| Bristowe Bros. | 10 | 10 |
| Blew-Jones & Mosenthal | 10 | 10 |
| Alfd. Howell | 10 | 10 |
| E. Blackborn | 10 | 10 |
| W. Asch | 10 | 10 |
| W. Kaulla | 10 | 10 |
| W. T. Morrison | 10 | 10 |
| Montagu, Oppenheim, and Co. | 10 | 10 |
| A. A. Marks | 10 | 10 |
| Snell and Swaffield | 10 | 10 |
| G. W. Dennis | 10 | 10 |
| A. J. Benjamin | 10 | 10 |
| Quilter, Balfour, & Co. | 10 | 10 |
| E. A. Ridsdale and Co. | 10 | 10 |
| A. J. Schwabe | 10 | 10 |
| H. Henderson | 10 | 10 |
| Sandeman, Clark, & Co. | 10 | 10 |
| Malcolm Cooke and Lemon | 10 | 10 |
| Oakley Bros. | 10 | 10 |
| G. Pember | 10 | 10 |
| P. Quirk | 10 | 10 |
| Byng, Foley, and Co. | 10 | 10 |
| Hunt, Cox, and Co. | 10 | 10 |
| Levi Cohen and Co. | 10 | 10 |
| Julian Joseph and Co. | 10 | 10 |
| M. Schwabacher & Co. | 10 | 10 |
| E. S. Marcus | 10 | 10 |
| H. J. Baker | 10 | 10 |
| Millar and Llewellyn | 10 | 10 |
| Cutcliffe, Ley, and McCulloch | 10 | 10 |
| H. Pearson and Co. | 10 | 10 |
| Wackerbath & Smith | 5 | 5 |
| Morison and Wood | 5 | 5 |
| R. H. Bristowe and Co. | 5 | 5 |
| C. G. Stretten | 5 | 5 |
| E. Cutbill | 5 | 5 |
| Palmer Brothers | 5 | 5 |
| E. Campbell | 5 | 5 |
| J. Hawkins | 5 | 5 |
| Goldberger & Lindeck | 5 | 5 |
| S. Lazarus | 5 | 5 |
| C. T. Pulley | 5 | 5 |
| F. H. Gibbes | 5 | 5 |
| Stanley Eagle | 5 | 5 |
| Hugh Campbell | 5 | 5 |
| Hy. Dunn | 5 | 5 |
| B. J. Angle | 5 | 5 |
| Samuelson and Co. | 5 | 5 |
| H. T. Brice | 5 | 5 |
| Walker and Ibbotson | 5 | 5 |
| Ramsden, Frisby, & Co. | 5 | 5 |
| C. Douglas Clarke and Co. | 5 | 5 |
| Wildy Bros. | 5 | 5 |
| J. H. Watts | 5 | 5 |
| Southard Gilbey | 5 | 5 |
| E. F. Thorpe and Tydd | 5 | 5 |
| A. J. Baker | 5 | 5 |
| C. Cotes | 5 | 5 |
| A. H. Roughton | 5 | 5 |
| Carr and Banks | 5 | 5 |
| Bradford and Paine | 5 | 5 |
| Jos. Procter | 5 | 5 |
| H. Sutherland | 5 | 5 |
| Murton and Adams | 5 | 5 |
| Hall and Co. | 5 | 5 |
| H. H. Barnes | 5 | 5 |
| Lumsden and Myers | 5 | 5 |
| Hodson, Jarrett, and Guscotte | 5 | 5 |
| J. E. Clayton | 5 | 5 |
| A. Chalk | 5 | 5 |
| F. Hastings | 5 | 5 |
| Baker, Dickinson, and Hardcastle | 5 | 5 |
| H. Lowry | 5 | 5 |
| H. Le B. Smith | 5 | 5 |
| Simons and Raincock | 5 | 5 |
| W. Seal | 5 | 5 |
| Tatham and Gibbs | 5 | 5 |
| R. Baxter | 5 | 5 |
| Emberson and Hall | 5 | 5 |
| T. H. Bower | 5 | 5 |
| Geo. Concanon | 5 | 5 |
| H. T. Peckham | 5 | 5 |
| Banks Bros. | 5 | 5 |
| Drake and Schwann | 5 | 5 |
| C. Paine | 5 | 5 |
| G. Whitehead & Chown | 5 | 5 |
| Scrutton and Sons | 5 | 5 |
| G. W. Dawes | 5 | 5 |
| J. Norbury | 5 | 5 |
| Nat. Morris | 5 | 5 |
| A. L. Reynolds | 5 | 5 |
| W. G. Woolston | 5 | 5 |
| Jno. Allen | 5 | 5 |
| B. A. W. | 5 | 5 |
| A. Johnson and Co. | 5 | 5 |
| E. S. Revett | 5 | 5 |
| C. H. Jackson | 5 | 5 |
| J. T. King | 5 | 5 |
| G. F. Garry | 5 | 5 |
| A. Kohn | 5 | 5 |
| A. Seligman | 5 | 5 |
| C. J. Whittington | 5 | 5 |
| A. C. Ionides | 5 | 5 |
| G. C. Howard | 5 | 5 |
| J. M. Pritchard & Co. | 5 | 5 |
| J. Friedberger | 5 | 5 |
| Hardy Bros. | 5 | 5 |
| Nathan and Rosselli | 5 | 5 |
| J. B. Concanon | 5 | 5 |
| A. S. G. Graves & Co. | 5 | 5 |
| Hon. K. P. Bouverie | 5 | 5 |
| Capel-Cure and Terry | 5 | 5 |
| J. G. Bone and Sons | 5 | 5 |
| Spurling and Skinner | 5 | 5 |
| Gray and Guy | 5 | 5 |
| Rowe and Pitman | 5 | 5 |
| C. H. Carlisle | 5 | 5 |
| G. H. and A. M. Jay | 5 | 5 |
| Harry Sopper | 5 | 5 |
| May and Hart | 5 | 5 |
| Walter Plimpton | 5 | 5 |
| Holloway and Platten | 5 | 5 |
| Roger Mortimer | 5 | 5 |
| W. P. Brown | 5 | 5 |
| De Boos and Davis | 5 | 5 |
| W. H. Hart | 5 | 5 |
| Anon | 5 | 0 |
| H. W. Lawson | 5 | 0 |
| R. Weguelin | 3 | 3 |
| A. G. Scrase | 3 | 3 |
| J. Norbury, jun. | 3 | 3 |
| A. C. Radmall | 3 | 3 |
| Robt. Matthews | 3 | 3 |
| R. Faulconer | 3 | 3 |
| C. Stanhope | 3 | 0 |
| A. J. D. | 2 | 2 |
| G. B. B. | 2 | 2 |
| Ferdinand Hess | 2 | 2 |
| Norman Paine | 2 | 2 |
| H. F. Medley | 2 | 2 |
| C. B. | 2 | 2 |
| W. H. | 2 | 2 |
| B. Tatham | 2 | 2 |
| A. O. Barnes | 2 | 2 |
| A. Fletcher | 2 | 2 |
| Tom Wilson | 2 | 2 |
| J. D. Maclure | 2 | 2 |
| Reynell and Stocken | 2 | 2 |
| Hon. A. E. Harbord | 2 | 2 |
| D. M. Scott | 2 | 2 |
| Lamb and Gold | 2 | 2 |
| F. Helyar | 2 | 2 |
| Morison and Lermitte | 2 | 2 |
| C. Mendel | 2 | 2 |
| Miss E. W. | 2 | 2 |
| F. Snoad | 2 | 2 |
| G. E. | 2 | 2 |
| C. L. Lewes | 2 | 2 |
| F. Donnison | 2 | 2 |
| F. W. Petch | 2 | 2 |
| C. H. Trimm | 2 | 2 |
| W. Busby | 2 | 2 |
| A. Cohen | 2 | 2 |
| W. J. Duncum | 2 | 2 |
| P. St. John Langton | 2 | 2 |
| Anon | 2 | 2 |
| Sidney Hellyer | 2 | 2 |
| B. B. | 2 | 2 |
| Joseph Blake | 2 | 2 |
| W. Godfrey | 2 | 2 |
| A. H. Hart | 1 | 1 |
| J. T. Illington | 1 | 1 |
| R. G. Blumenthal | 1 | 1 |
| J. H. | 1 | 1 |
| J. M. | 1 | 1 |
| G. S. | 1 | 1 |
| S. J. Notley | 1 | 1 |
| C. E. McKenna | 1 | 1 |
| A. A. Ellis | 1 | 1 |
| Received too late for insertion | 97 | 18 |
| Total | £3,400 | 0 |

